# Does the Potential for Chaos Constrain the Embryonic Cell-Cycle Oscillator?

**DOI:** 10.1371/journal.pcbi.1002109

**Published:** 2011-07-14

**Authors:** R. Scott McIsaac, Kerwyn Casey Huang, Anirvan Sengupta, Ned S. Wingreen

**Affiliations:** 1 Lewis-Sigler Institute for Integrative Genomics, Princeton University, Princeton, New Jersey, United States of America; 2 Graduate Program in Quantitative and Computational Biology, Princeton University, Princeton, New Jersey, United States of America; 3 Department of Bioengineering, Stanford University, Stanford, California, United States of America; 4 Department of Physics & Astronomy, Rutgers University, Piscataway, New Jersey, United States of America; 5 BioMAPS Institute for Quantitative Biology, Rutgers University, Piscataway, New Jersey, United States of America; 6 Department of Molecular Biology, Princeton University, Princeton, New Jersey, United States of America; Medical College of Wisconsin, United States of America

## Abstract

Although many of the core components of the embryonic cell-cycle network have been elucidated, the question of how embryos achieve robust, synchronous cellular divisions post-fertilization remains unexplored. What are the different schemes that could be implemented by the embryo to achieve synchronization? By extending a cell-cycle model previously developed for embryos of the frog *Xenopus laevis* to include the spatial dimensions of the embryo, we establish a novel role for the rapid, fertilization-initiated calcium wave that triggers cell-cycle oscillations. Specifically, in our simulations a fast calcium wave results in synchronized cell cycles, while a slow wave results in full-blown spatio-temporal chaos. We show that such chaos would ultimately lead to an unpredictable patchwork of cell divisions across the embryo. Given this potential for chaos, our results indicate a novel design principle whereby the fast calcium-wave trigger following embryo fertilization synchronizes cell divisions.

## Author Summary

Cell divisions across an embryo occur in rapid synchrony - like clockwork - starting within minutes of fertilization. How does an embryo achieve this remarkable uniformity? Simple diffusion is too slow: typical proteins diffuse with a rate of 10 *µ*m^2^/s, requiring nearly 14 hours to traverse a 1 mm embryo. An exciting idea is that the embryo is an active medium, much like the heart where pulses of electrical activity result in organized contractions. However, just as the heart can have arrhythmias, our model predicts that oscillations in the embryo can become chaotic. What would be the biological consequences of this behavior? How do embryos avoid chaos? Our work provides potential answers to these questions: Chaos would lead to an unpredictable patchwork of cell divisions across the embryo - clearly a fatal defect in development. To avoid chaos then, we predict that cell-cycle oscillations need to be triggered throughout the embryo at almost precisely the same time. The threat that chaos will mar development therefore explains the mystery of why embryos universally employ a fast calcium wave to trigger cell-cycle oscillations. In this way, developing organisms get the synchronizing benefits of an active medium without suffering the destructive consequences of chaotic arrhythmias.

## Introduction

The early stages of embryo development require the integration of two separate design principles: a network that generates sustained oscillations and a control mechanism that produces robust spatial synchronization. While considerable research has addressed oscillatory behavior in different biological contexts [1], the question of how embryos are synchronized across large spatial dimensions has yet to be addressed. Although simple diffusion alone is insufficient to communicate the stage of the cell cycle over typical embryonic length scales (0.1–1 mm), it has been proposed that the combination of certain molecular species in a developing embryo may be regarded as an active medium [2]. Other biological examples of active media include the transmission of fast-moving cAMP waves by the single-celled organism *Dictyostelium discoideum* [3] to allow for aggregation in response to nutrient deprivation, and the heart, where waves of electrical activity lead to organized contractions [4]. Active media allow for the transmission of information over large length scales at rates far greater than allowed by simple diffusion. Yet the existence of an active medium coupled to an oscillatory system does not guarantee spatial synchrony: active media can also give rise to complex spatial patterns, including chaos [5]. Broadly speaking, chaos describes behaviors in which slight perturbations to initial conditions result in highly divergent, unpredictable outcomes. If the embryo indeed functions as an active medium during development, what guarantees that oscillations will be uniform and not spatially patterned or even chaotic?

In vertebrates, oogenesis begins with the production of immature oocytes, which arrest in the prophase of meiosis I [6]. These oocytes undergo maturation in response to progesterone, and, in many vertebrates (including frogs, mice, and humans) [7], progress to and arrest in metaphase of meisosis II [8], [9]. In the egg of the African clawed frog *Xenopus laevis*, cell division is driven by a molecular clock that modulates Cdk1 activity through a combination of positive and negative feedback [10], [11]. Prior to fertilization a cytoplasmic activity termed cytostatic factor (CSF) [12] interrupts the negative feedback [13] by inhibiting the anaphase-promoting complex (APC) [7], [14], a ubiquitin ligase that targets mitotic cyclins for degradation [15], [16], thereby keeping the egg arrested in metaphase. Once in metaphase of meiosis II, the oocyte is mature and able to be fertilized. Fertilization results in a rapid, transient increase in intracellular calcium, beginning at the site of sperm entry, traveling across the egg, and terminating at the antipode [17]. The propagation of this calcium wave [17], [18], [19] leads to the destruction of CSF [20], [21], [22] and the onset of synchronous nuclear divisions. The diameter of an *X. laevis* egg is 1–1.2 mm and the calcium wave speed has been experimentally measured to be 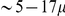
m/s (speed varies slightly depending on location) [17], so that it takes just a few minutes for the wave to travel across the egg. By inactivating CSF, the calcium wave initiates cell-cycle oscillations and the *X. laevis* egg subsequently undergoes 12 rounds of synchronous cell divisions in 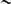
8 hours [23].

**Figure 1:**
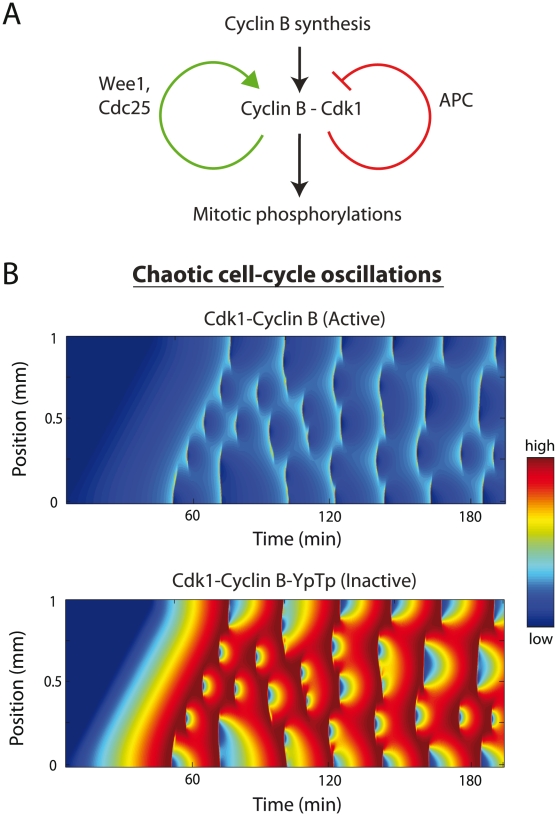
Diffusive spread of cell-cycle activity. (A) The cell-cycle is driven by a combination of positive and negative feedback loops, which modulate Cdk1 activity through phosphorylation/dephosphorylation. (B) 3.33-hour simulation where cell-cycle activity spreads across a 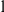 mm embryo. In the spreading model, oscillations initially occur in only 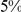 of the embryo (*i.e.*, APC is initially assumed to be active only between 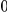 and 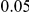 mm in a 1 mm long embryo). Through diffusion, oscillations are able to spread across the embryo via local activation of APC once a local critical threshold of overall Cdk1 activity is reached. Each species is initially at its pre-fertilization concentration, with the activity threshold chosen to be when Cyclin B-Cdk1-Tp reaches 4× its initial value. The species plotted in the top panel is Cyclin B-Cdk1 (active) and that plotted in the bottom panel is Cyclin B-Cdk1-YpTp (inactive). In the inactive complex, Cdk1 is phosphorylated at both Threonine-14 and Tyrosine-15.

*X. laevis* is a model system for studying the embryonic cell-cycle network. Developing embryos have relatively simple cell cycles, each consisting of two phases, interphase and metaphase, which are characterized by low and high levels of Cdk1 activity, respectively. How does the cell-cycle network toggle between these two levels of Cdk1 activity? Experimentally, Cdk1 activity has been measured in *X . laevis* egg extracts in the presence of controlled amounts of a non-destructible form of cyclin, 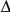
65-cyclin B1 [11]. The sharp, hysteretic response of Cdk1 activity to changes in 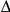
65-cyclin B1 concentration indicates an underlying bistability of the network. To support this conclusion, Pomerening *et al.* [11] developed a numerical model of the embryonic cell-cycle network in which Cdk1 activity is increased by dual positive feedbacks (active Cdk1 activates its activator Cdc25 and inactivates its inhibitor Wee1), while Cdk1 activity is reduced by a single negative feedback (active Cdk1 activates APC via Plx1) (Fig. 1A, S1). Elimination of the dual positive positive feedbacks results in damped, “sluggish” oscillations of Cdk1 activity, and thus, defective cell cycles [11]. Qualitatively, the system resembles a simple relaxation oscillator in which fast positive feedback leads to two stable states, and slow negative feedback leads to oscillations between these two states. Tsai *et al.* [24] argued persuasively that oscillators of this type provide a good fit to the biological requirements of the cell cycle, including a freely tunable frequency and robust, large amplitude oscillations sufficient to drive mitotic phosphorylations.

## Results

To better understand the requirements for spatial synchrony in the embryo, we have generalized the model of Pomerening *et al.* [11] to a spatially extended system, including free diffusion of proteins (Text S1). Typical *in vivo* diffusion constants vary by protein and environment, but have been measured to be in the range 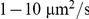 [25], [26], [27]. In the cytoplasm of a *D. melanogaster* embryo, 40 kDa fluorescently-labeled dextran molecules have a measured diffusion constant of 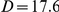 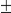
1.8 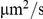, providing an effective upper bound for typical protein diffusion [28]. The Stokes-Einstein relation predicts that 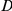 is relatively insensitive to mass (Text S1), and all of the *X. laevis* cell-cycle proteins have similar molecular weights. We therefore set 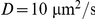 for each species in our simulations (except where noted). An appropriate choice of initial conditions was obtained in two steps. First, the ODE model (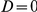) was integrated to reach limit-cycle oscillations and metaphase was determined to occur at the peak of active Cdk1. Second, starting from metaphase, the ODE model was solved with 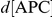 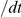 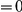 to mimic the activity of CSF, yielding pre-fertilization steady-state concentrations for each species.

### Chaos in the cell-cycle oscillator

Within the picture of the embryo as an active-medium, one might expect that fertilization would initiate cell-cycle oscillations at a particular point with activity simply spreading across the embryo like fire spreading through a forest. A mechanism of this type was originally proposed by Novak and Tyson [29]. To test this hypothesis, we consider a 1D embryo in metaphase, and initiate oscillations at one end of the embryo. We observe that oscillations can spread across the embryo by diffusion if we let oscillations at each point commence once a local critical threshold of overall Cdk1 activity is reached (in Fig. 1B, the Cdk1 activity threshold was chosen as 4× its initial value). We have displayed the concentration of both the active Cyclin B-Cdk1 and the doubly phosphorylated, inactive Cyclin B-Cdk1-YpTp for visual comparison in Fig. 1B. Note that sharp increases in active Cyclin B-Cdk1 occur as Cyclin B-Cdk1-YpTp sharply decreases (Fig. 1B, S3). We will display the concentration of the inactive Cyclin B-Cdk1-YpTp in subsequent figures, but we note that all protein species display similar features (Fig. S2).

In Fig. 1B, oscillations spread across the embryo at a fixed velocity, taking 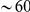 minutes to reach the other end. This demonstrates that the set of cell-cycle equations admits traveling wave solutions. However, a chaotic pattern arises almost immediately following the progression of this initial wave and persists throughout the 200-minute simulation (in this article, we refer to chaos as the non-uniform, unpredictable occurrence of cell-cycle oscillations in the embryo). In conclusion, we find that the spreading scheme does not guarantee robust spatio-temporal synchronization; in fact, the result is chaotic oscillations. This observation motivated us to explore the role of the rapid calcium wave in synchronizing cell-cycle oscillations and preventing the emergence of chaos.

### Fast calcium waves result in robust cell-cycle synchronization

**Figure 2:**
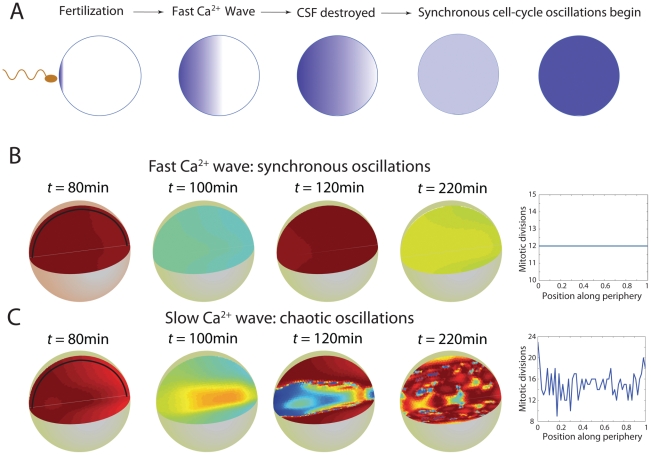
Modeling the cell-cycle in developing embryos. (A) Schematic of early development. Across species, fertilization results in a fast calcium wave that sweeps across the embryo. In *X . laevis*, the result is the destruction of CSF, the consequent restoration of APC-mediated negative feedback, and the subsequent initiation of synchronous nuclear divisions. (B,C) Cell-cycle simulations for spherical embryos with diameters of 1 mm. The fast and slow calcium waves have speeds of 0.36 and 0.125 mm/min, respectively. The number of mitotic divisions that occur in 8 hours along the black lines in the first panel are shown at right.

In *X. laevis* embryos prior to fertilization, CSF activity inhibits the activity of APC, the negative regulator of Cyclin B-Cdk1 levels. As the post-fertilization calcium wave sweeps across the embryo as a plane wave, CSF activity is progressively eliminated and APC is able to target cyclins for destruction. To mimic this behavior, we performed simulations in which APC becomes active (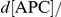 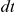 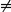 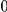) at a particular point in space as soon as the calcium wave reaches that point. To determine the relationship between the speed of the calcium wave and the embryo's ability to achieve synchronous cell cycles, we carried out simulations using an embryo-like spherical geometry with a diameter of 1 mm (Fig. 2B,C). For fast calcium waves (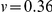 mm
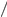
min = 6 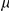
m/s), the system subsequently achieves robust, synchronous oscillations (Fig. 2B). However, for slower waves (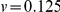 mm
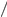
min
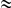
2.08 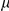
m/s, still significantly faster than the propagation of oscillations in Fig. 1B), while the initial oscillations appear almost synchronous, after a few cycles the system gives way to full-blown chaos (Fig. 2C). What would such chaotic oscillations mean for the developing embryo? In the plot at the right of Fig. 2C, we show the total number of nuclear divisions that would occur by 8 hours at the surface of the embryo, defining a division event to occur when the concentration of Cyclin B-Cdk1-YpTp species increases past its half-maximum for homogeneous oscillations (the number of mitotic events is relatively insensitive to the choice of threshold). The patchy pattern of total divisions contrasts sharply with the exact, uniform 12 divisions in 8 hours for synchronous oscillations (see Figs. 2B, S4, S5, S6).

### Quantifying spatio-temporal chaos in 1D embryos

**Figure 3:**
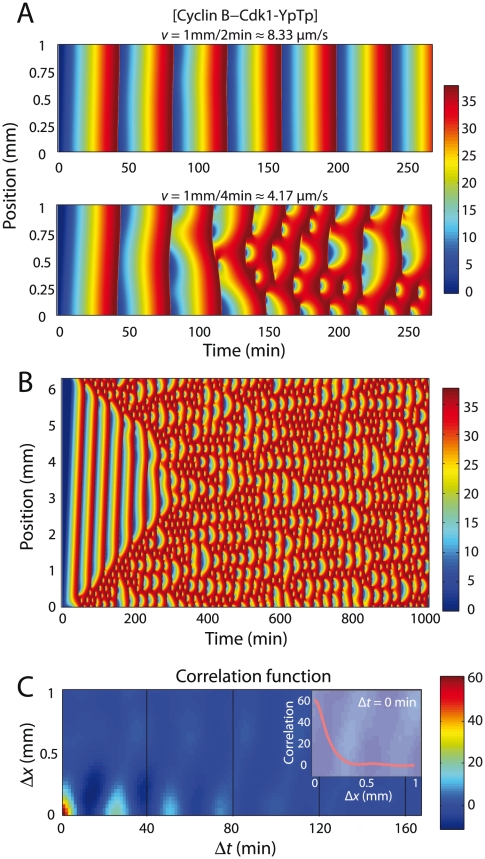
Cell-cycle equations exhibit chaotic behavior with extreme sensitivity to initial conditions. (A) Simulations with two different calcium wave speeds in 1 mm embryos, with colormap showing the concentration of doubly phosphorylated Cyclin B - Cdk1 complex. Simulation cell is 1D with no flux boundary conditions. (B) Simulation in a large (6 mm) 1D embryo with periodic boundary conditions. (C) The space-time correlation function is computed using the mean-centered values in (B) between 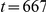 min. and 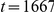 min. (inset) The correlation length is computed to be 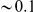 mm at 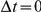.

**Figure 4:**
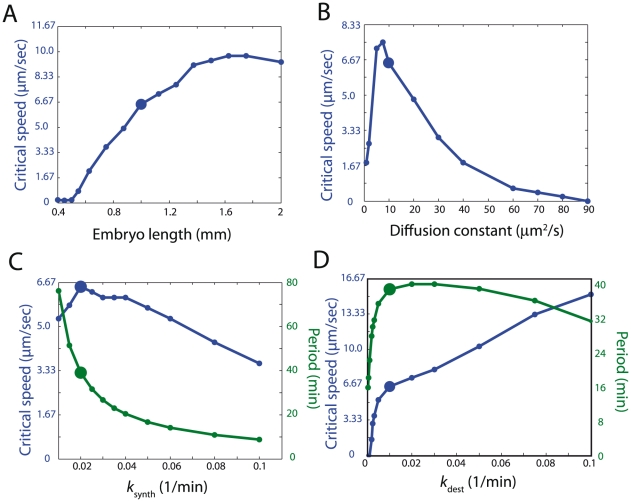
Sensitivity analysis of cell-cycle equations. (A–D) The critical calcium wave speed, 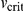, and the period, 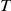, are computed for different values of the (A) embryo length, (B) protein diffusion constant, (C) rate of cyclin synthesis 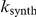, and (D) rate of APC activity 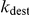.

To further probe the relationship between calcium wave speed and synchrony, we conducted simulations of 1D embryos. The 1D simulations fully capture the oscillatory behavior while better revealing key features of the chaotic oscillations. Remarkably, in both 1D and 3D we observe a very small ratio of 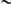
1/10 between the critical time for the wave to propagate, 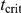, and the cell-cycle period, 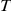 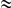
40 min. For a 1D embryo of length 1 mm, we find the critical calcium wave speed is 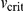 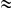
1 mm/2.5 min
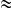
6.7 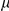
m/s (Figs. 3A, 4A–D). For a 3D embryo with the same spherical geometry as in Fig. 2C, 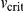 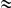
1 mm/4.5 min
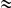
3.7 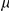
m/s, slightly slower than the experimentally measured range. To better characterize the chaotic behavior, we also simulated a 6 mm embryo, with periodic boundary conditions, triggered by a slow-speed calcium wave (Fig. 3B). Figs. 3A (bottom panel) and Fig. 3B highlight a characteristic length scale of spatial variations and an effective frequency doubling of the oscillations. To quantify these observations, we computed the space-time correlation function in the chaotic regime for the doubly phosphorylated Cyclin B-Cdk1 species (Fig. 3C). The correlation function displays a short spatial correlation length (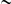
0.1 mm) typical of spatial chaos, but preserves a weak signature of frequency-doubled oscillations over long times. The distribution of sizes of chaotic features is roughly independent of embryo length or boundary conditions. In the *X. laevis* embryo, fertilization occurs at a single position along the boundary. In our simulations, the future dynamics only depend on this position when in the chaotic regime. Nevertheless, chaotic features with a characteristic length scale emerge consistently in our model for both periodic and no-flux boundary conditions for the full range of embryo sizes we consider.

### Sensitivity analysis of cell-cycle equations

The critical calcium wave speed is the minimum speed required to achieve synchronous oscillations. How does the critical wave speed depend on embryo size, diffusion, and cell-cycle parameters? We observe chaos over a range of embryo lengths, and find that larger embryos require faster calcium waves to enforce synchrony (Fig. 4A). Intriguingly, there is a minimum size around 0.4 mm below which even extremely slow-speed waves eventually synchronize the embryo (Fig. S8). As expected, increasing the diffusion constant above 10 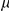
m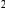/s reduces 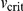 (Fig. 4B). Curiously, 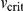 also decreases as 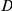 drops below 5 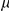
m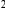/s. Two key cell-cycle parameters are the rate of cyclin production, 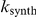, to which the oscillation period 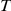 is inversely proportional (Fig. 4C), and the rate at which cyclins are destroyed due to APC, 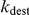. As 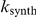 increases we find a maximum in 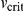 near the wild-type period (Fig. 4C). As 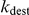 increases, we find that the critical wave speed monotonically increases (Fig. 4D). In other words, increasing APC activity makes the embryo less tolerant to slow wave speeds.

### Cellularization and chaos

**Figure 5:**
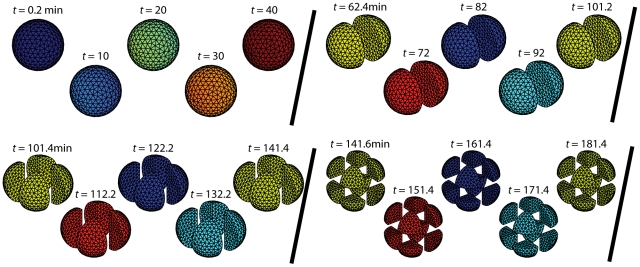
Cell-cycle simulations with cellularization. The embryo is a 1 mm diameter sphere and the calcium wave speed is 1 mm/4 min
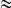
4.17 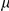
m/s. Mitotic divisions are taken when Cyclin B-Cdk1-YpTp reaches half its concentration for uniform oscillations, and are modeled by a no-flux boundary dividing a cell into two equal volumes. By this metric, the first division occurs after 62 minutes, and each subsequent division occurs at 39.2-minute intervals. Solid diagonal lines indicate cellular divisions.

**Figure 6:**
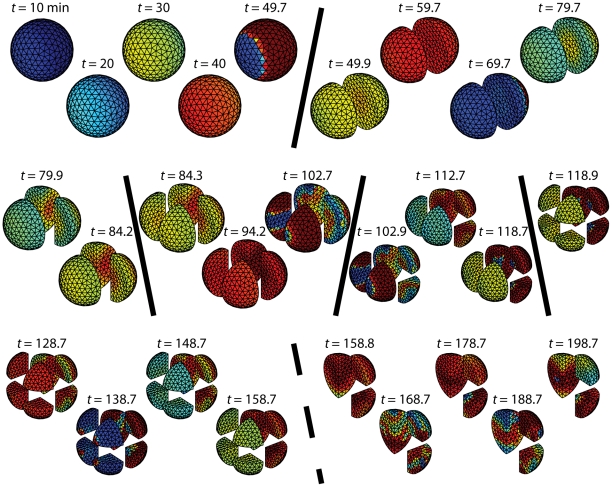
Cellularization does not prevent chaos for a slow Ca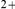 wave Simulations as in Fig. 5, but for a calcium wave speed of 1 mm/10 min
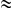
1.67 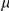
m/s. The solid diagonals indicate at least one cell division. Following the dashed line, even when the simulation continues for longer times, the displayed portions do not divide.

Given that smaller embryos are less sensitive to chaos, we considered whether cellularization would be sufficient to ensure cell-cycle synchrony. To address this question, we performed simulations in a spherical embryo that explicitly include cellularization (Fig. 5). Specifically, when the average level of Cyclin B-Cdk1-YpTp increased above the level halfway between the minimum and maximum values of a limit-cycle oscillation, a no-flux boundary condition was introduced to divide the parent cell into two daughter cells, each of equal volume. For three rounds of cell division triggered by a calcium wave speed of 1 mm/4 min
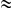
4.17 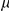
m/s, we observed complete synchrony of cell divisions along with a homogeneous distribution of cell-cycle components (Fig. 5). However, when the calcium wave speed was slowed to 1 mm/10 min
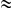
1.67 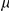
m/s (less than half the average speed of experimentally measured calcium waves) we observed the phenotype of chaos: an unpredictable patchwork of cell divisions across the embryo (Fig. 6). The first cell division resembles that of Fig. 5, but the spatial variations in concentrations are such that the cell distal to the point of fertilization divides 5 minutes before the proximal half. Subsequent divisions become increasingly unpredictable with chaotic spatial patterns even within the smaller cellularized compartments. In addition to uncoordinated divisions with varied periods, some cells ceased dividing entirely during the simulation (Fig. 6, bottom right). Hence, cellularization by itself does not appear to be enough to prevent chaos if cell-cycle oscillations are triggered by a slow calcium wave.

## Discussion

Previous work has established the role of intracellular calcium in destroying CSF activity, yet the necessity of a *fast* post-fertilization calcium wave has yet to be addressed. Intuitively, one might have imagined that, following fertilization, cell-cycle oscillations would simply be nucleated at the site of sperm entry, and this activity would spread across the embryo, resulting in synchronous divisions. In that case, what would be the need for a calcium wave? Our work provides a possible answer to this puzzle: yes, activity could spread via diffusion from one point but the result would be chaos, not synchronized oscillations, leaving in its aftermath unpredictable variations in the number of nuclear divisions in different parts of the embryo (Fig. 1B).

Instead, the fast calcium wave provides a robust solution for synchronizing embryonic cell cycles across the range of vertebrates from the 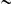
1 mm eggs of many amphibians and fish down to the 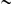
100 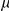
m eggs in humans, mice, and other mammals [30]. Interestingly, for these latter smaller embryos, activity spread due to simple diffusion through an active medium could lead to synchrony (Fig. S9), raising the question of whether conservation of the post-fertilization calcium wave across vertebrates is due to heredity or to additional functional constraints.

For example, is there a constraint on the critical calcium wave speed due to the cell-cycle period? Though the occurrence of a sperm-entry-induced calcium wave followed by rapid nearly-synchronous cell divisions is widely conserved, the molecular details underlying this process vary by species. We have shown that the critical calcium wave transit time in *X. laevis* is 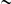
1/10 the cell-cycle period (Fig. S8). When the cell-cycle period is reduced to 10 minutes, as in *D. melanogaster*, the critical wave transit time is 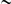
1/2 the cell-cycle period (Fig. S10). Going through the cell-cycle quickly, therefore, appears to allow for calcium waves whose transit times are larger fractions of the period. Though the calcium wave speed has not been explicitly measured in *D. melanogaster*, fertilization waves are typically 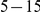 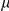
m/s in non-mammals [30], consistent with the requirements for synchrony.

Embryonic cells of *Xenopus laevis* spend 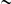
20–30 minutes in interphase, during which they replicate their DNA. Duplicated DNA pairs, called sister chromatids, must then be separated before cells can divide. Since we observe a frequency doubling of the 1D chaotic oscillations, one prediction is that chaos would result in cells not having sufficient time to replicate and separate chromatid pairs, thereby halting cell division altogether.

By performing simulations with a slow calcium wave in a 3D embryo-like geometry that explicitly includes cell division, we show that while some cells may continue to divide with wild-type periods despite the emergence of chaos, others will halt since they can no longer go through complete cell cycles (Fig. 6). Cells cease to divide from the unequal partitioning of proteins between daughter cells and this leads to the predicted phenotype of chaos: a patchwork of cell division where some cells cease dividing altogether.

Biophysical constraints can vary by species. For example, while the embryo of *D. melanogaster* is a syncytium in which the nuclei are coupled by a single cytoplasm, that of *X. laevis* has fully formed cells. Why have a syncytium? Clearly, it is not to establish synchrony, as one might have previously expected. Alternatively, why have cellularization? We have shown that cellularization is not sufficient to ensure synchronization. Intriguingly, for a slow calcium wave, there is a loss of phase coherence of cell-cycle components within individual cells, and this appears, as mentioned above, to halt cell division altogether in certain cases (Fig. 6). Fast waves result in phase coherence, thereby enforcing synchrony (Fig. 5). Our work suggests, therefore, that the presence of a syncytium or cellularization results from functional necessities independent of early synchronized cell divisions.

Our further analyses probed how central parameters in the cell-cycle model affect the establishment of synchrony. The protein diffusion constant 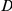 we chose for our simulations was 10 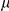
m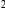/s. A sensitivity analysis of cell-cycle parameters demonstrated that this choice of 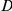 is in the worst range for the embryo (Fig. 4B). A possible explanation is that organisms have little control over protein diffusion constants, so selection acts primarily by adjusting relative abundances or activities of cell-cycle components, since tuning of these features can be achieved by simple mutations. Yet we also find that kinetic parameters are not tuned to optimal values for robust synchronization. For example, our model predicts that decreasing APC activity would allow the embryo to tolerate slower wave speeds (Fig. 4D). In this case, there are likely other determinants driving the selection of APC activity independent of achieving synchronous cell divisions post-fertilization. Importantly, the sensitivity analysis demonstrates that our central results do not require fine tuning of parameters. In conclusion, we have established the fragility to chaos of the embryonic cell-cycle oscillator in spatially extended systems, thereby motivating the fast post-fertilization calcium wave as a reliable means to synchronize nuclear divisions.

## Materials and Methods

### 1D simulations

The full set of cell-cycle equations from Ref. [24] was solved using a second-order finite difference scheme, where diffusion was accounted for with either the explicit FTCS (Forward-Time Central-Space) method or the implicit Crank-Nicolson method. The calcium-wave was implemented by modifying the reaction-diffusion equation for the concentration of active APC, 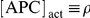:
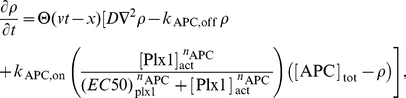
(1)where 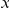 is the projected distance along the central axis from the fertilization point, 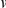 is the calcium wave speed, and 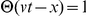 for 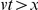. For all 1D simulations in the main and supplementary texts, the time step was 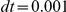 s and the spatial grid spacing was 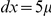
m. The critical calcium-wave speed 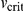 was determined by examining the synchronicity between the left and right half of the embryo over a 12-hour simulation.

### 3D simulations

Grids were generated using the distmesh collection of functions in Matlab [31]. For the simulations in Figs. 2 and S5, S6, S7, we first generated a grid of triangles covering a semicircle or semiellipse and then revolved these regions around the central axis to produce a sphere. This approximation to the full 3D geometry is equivalent to the assumption of fast diffusion in the azimuthal direction around the central axis, and allowed us to perform simulations with the same time step and similar grid spacings (average spacing between triangle vertices was 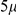
m) as in our 1D simulations. For the simulations in Figs. 5 and 6, we generated 3D grids of tetrahedra where the vertices that lie along the division planes defined a flat surface. To manage computational expense, the average spacing between vertices was 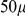
m.

Diffusion was accounted for using custom C++ software utilizing a biconjugate gradient sparse matrix solver and the Crank-Nicolson method. Upon cellularization, a no-flux boundary condition was implemented along the surface separating the two daughter cells.

### Correlation function

Given a species 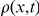, we define 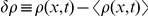, where 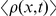 is the average value of 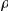 computed over both time and space. The correlation function in Fig. 3 for a particular pair 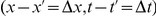 is given by
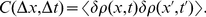
(2)


## Supporting Information

Figure S1 **The cell-cycle oscillator contains negative & dual positive feedbacks.** (Negative Feedback) The presence of the complex Cyclin-Cdk1-Tp results in the activation of Plx1, which phosphorylates and activates APC. Active APC then destroys cyclin B and Cyclin-Cdk1 complexes. (Dual Positive Feedbacks) The presence of Cyclin-Cdk1-Tp results in the activation of the Cdk1-activator, Cdc25, and the inactivation of the Cdk1-inhibitor, Wee1.
(EPS)

Figure S2 **Kymographs of all nine cell-cycle species from the simulations in** **Fig. 1B** **.**
(EPS)

Figure S3
**Comparing kinetic profiles of active and inactive Cyclin B-Cdk1.** The cell-cycle equations with 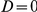 are integrated forward in time with a 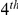-order adaptive-step Runge-Kutta solver. Cyclin B-Cdk1 (active) is shown in the top panel and Cyclin B-Cdk1-YpTp (inactive) is shown in the bottom panel. In this simulation, the rate of cyclin synthesis, 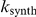, is 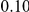, corresponding to a cell-cycle period of 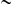
9 minutes.(EPS)

Figure S4
**Chaotic oscillations lead to spatial variation of mitotic divisions.** (A) 8-hour simulation of a 1D embryo of length 1 mm for a Ca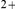 wave speed of 1 mm/min. (B) Spatial dependence of the total number of mitotic divisions in (A) after 8 hours. At each position along the embryo, a mitotic division is determined to occur when the concentration of Cyclin B-Cdk1-Yp-Tp increases past its half-maximum value. (C) Same as (A) but for a Ca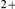 wave speed of 1 mm/6 min 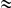
2.8 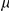
m/s. (D) The total number of mitotic divisions in (C) after 8 hours.(EPS)

Figure S5
**Cell-cycle simulations for prolate embryos with major and minor axes of 1 and 1/2 mm.** The slow and fast Ca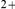 waves have speeds of 0.17 and 0.25 mm/min, respectively. The number of mitotic divisions that occur along the white and black lines in the first panel are shown at right. The critical Ca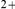 wave speed, 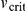, for this particular geometry is 1 mm/6 min 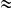
2.8 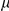
m/s.(EPS)

Figure S6 **Variability in the number of mitotic divisions within a prolate embryo.** The total number of mitotic divisions throughout a crossection of the chaotic prolate embryo in Fig. S3.
(EPS)

Figure S7
**Chaotic patterning in a spherical embryo.** Cell-cycle simulations for a spherical embryo with a diameter of 1 mm. The Ca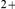 waves has a speed of 1 mm/6 min 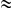
2.8 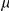
m/s.(TIF)

Figure S8
**Very slow-speed waves achieve spatial synchronization in short embryos.** 26.67 hour simulation where activity spreads across a 300 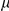
m embryo for a Ca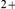 wave speed of 1 mm/100 min. After an initial period of spatial variation, the embryo settles into a stable synchronized oscillation pattern.(EPS)

Figure S9 **Diffusive spread of cell-cycle activity in 0.1 mm embryo results in synchrony.** 3.33-hour simulation where cell-cycle activity spreads across an embryo, as in Figure 1 of the main text.
(EPS)

Figure S10
**Critical Ca** 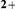 **wave speeds and transit times versus** 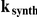 **.** (Green curve) Critical speed for chaotic oscillations as a function of the cyclin synthesis rate 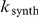. (Blue curve) Ratio of critical transit time, 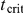, to oscillation period, 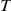, as a function of 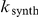. The critical transit time, 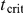, is computed by dividing the length of the embryo, 1 mm, by the critical Ca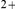 speed, 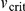.(EPS)

Text S1 **Supporting information.**
(PDF)
