# Supplementary figures and images for: Does the Potential for Chaos Constrain the Embryonic Cell-Cycle Oscillator?
Source: PLoS Comput Biol. 2011 Jul 14;7(7):e1002109. doi: 10.1371/journal.pcbi.1002109 (PMC3136431; doi:10.1371/journal.pcbi.1002109)

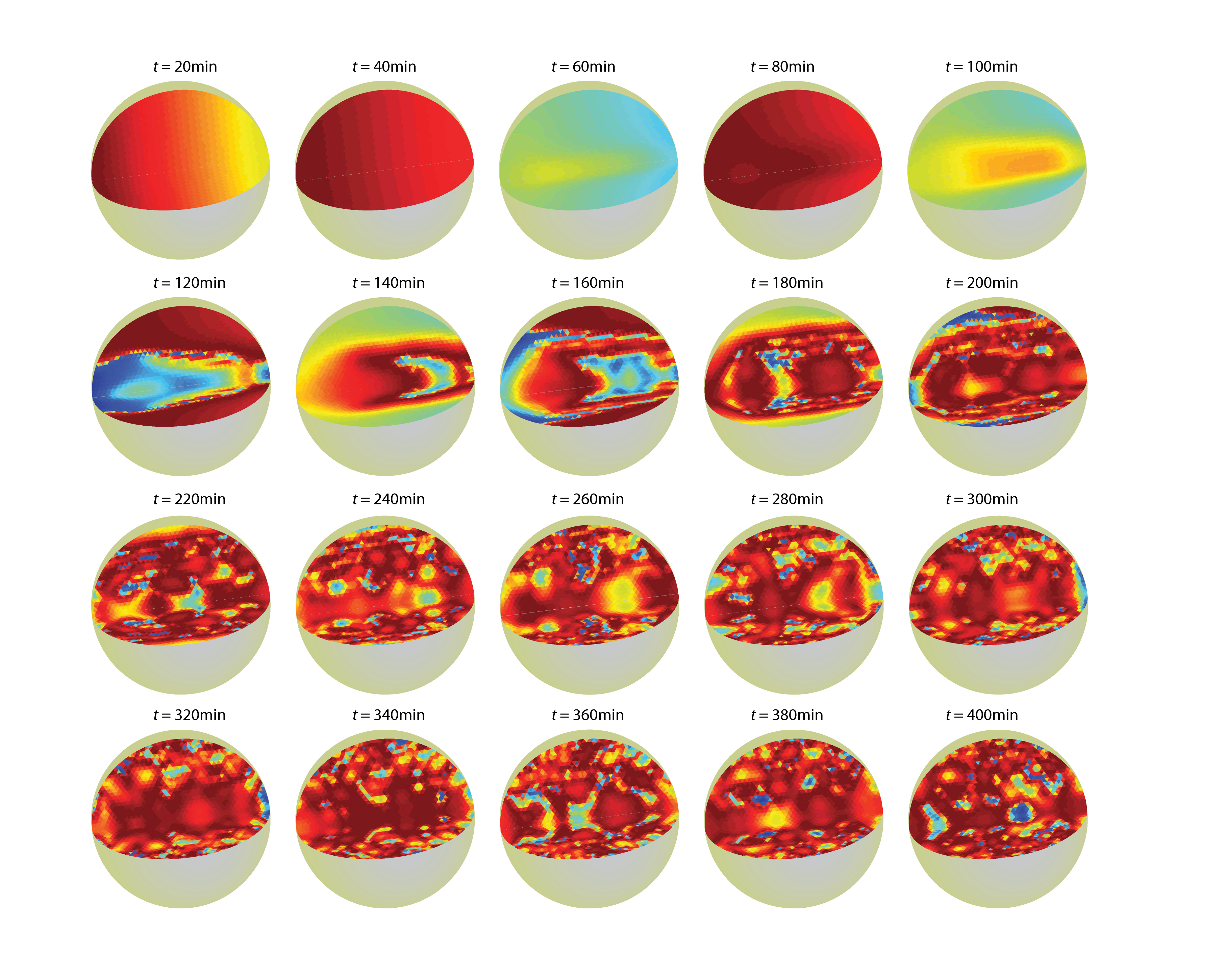

Supplement: Figure S7 — Chaotic patterning in a spherical embryo. Cell-cycle simulations for a spherical embryo with a diameter of 1 mm. The Ca waves has a speed of 1 mm/6 min 2.8 m/s. (TIF) [file pcbi.1002109.s007.tif]
