# Supplementary material for: Does the Potential for Chaos Constrain the Embryonic Cell-Cycle Oscillator?
Source: PLoS Comput Biol. 2011 Jul 14;7(7):e1002109. doi: 10.1371/journal.pcbi.1002109 (PMC3136431; doi:10.1371/journal.pcbi.1002109)
Supplement: Text S1 — Supporting information. (PDF) [file pcbi.1002109.s011.pdf]

Text S1

Does the potential for chaos constrain the embryonic cell-cycle oscillator?

R. Scott McIsaac[1,2,*], Kerwyn Casey Huang[3,*], Anirvan Sengupta[4,5], Ned S. Wingreen[1,6,†]

[1]Lewis-Sigler Institute for Integrative Genomics, Princeton University, Princeton, NJ 08544

[2]Graduate Program in Quantitative and Computational Biology, Princeton University, Princeton, NJ 08544

[3]Department of Bioengineering, Stanford University, Stanford, CA 94305

[4]Department of Physics & Astronomy, Rutgers University, Piscataway, NJ 08854

[5]BioMAPS Institute for Quantitative Biology, Rutgers University, Piscataway, NJ 08854

[6]Department of Molecular Biology, Princeton University, Princeton, NJ 08544

[*]These authors contributed equally to this work.

[†]To whom correspondence should be addressed;

Department of Molecular Biology

119 Lewis Thomas Laboratory

Princeton, NJ 08544 USA;

E-mail: wingreen@princeton.edu

Phone: (609)-258-8476

Cell-cycle model

The most current model of the embryonic cell cycle in *X. laevis* was derived by Pomerening *et al.* [1] In the current study, we extended this model to include a spatial component. Simulations were performed in 1D and 3D geometries, with all species able to diffuse.

At a qualitative level, oscillations in Cdk1 activity are driven by dual fast positive feedbacks and a slower negative feedback. The period of the oscillations is determined by the rate of cyclin B synthesis [2]. Cdk1 binds cyclin B (forming Cyclin B-Cdk1), and, upon activation, the active Cyclin B-Cdk1 complex phosphorylates the mitotic substrates that transition the cell from interphase to M-phase. The transition from M-phase back to interphase is driven by anaphase-promoting complex (APC)-mediated polyubiquitination of cyclin B [3, 4, 5].

In the Pomerening *et al.* [1] model, the dynamics of cyclin B and the phosphorylated/ dephosphorylated states of the Cyclin B-Cdk1 complex are explicitly modeled. Phosphorylation at Tyr-15 results in inactivation of the Cyclin B-Cdk1 complex; namely, Cyclin B-Cdk1-Yp and Cyclin B-Cdk1-YpTp (also phosphorylated at Thr-14) are inactive. The complex Cyclin B-Cdk1-Tp is phosphorylated at Thr-14 and lacks phosphorylation at Tyr-15.

To achieve negative feedback, the presence of the complex Cyclin B-Cdk1-Tp results in activation of the polo-like kinase, $\text{Plx1}|_{\text{act}}$. $\text{Plx1}|_{\text{act}}$ then phosphorylates and activates the anaphase-promoting complex, $\text{APC}|_{\text{act}}$, which destroys cyclins and Cyclin B-Cdk1 complexes. Dual positive feedbacks are achieved through the Cyclin B-Cdk1-Tp-mediated activation of the Tyr-15 phosphatase, $\text{Cdc25}|_{\text{act}}$, and the Cyclin B-Cdk1-Tp-mediated inactivation of the Tyr-15 kinase $\text{Wee1}|_{\text{act}}$.

Central parameters in the model include k_{synth}, the rate of cyclin B synthesis; k_{dest}, the rate at which active APC destroys cyclins; and r, the strength of the positive feedbacks. The active forms of Cdc25 and Wee1 ($\text{Cdc25}|_{\text{act}}$ and $\text{Wee1}|_{\text{act}}$) have phosphatase and kinase activities given by k_{cdc25} and k_{wee1}, respectively. The basal activities of Cdc25 and Wee1 are k_{wee1}/r and k_{cdc25}/r, where the parameter r is the fraction of phosphatase/kinase activity in interphase versus M-phase. When $r = 1$, the model becomes negative-feedback-only.

$$
\begin{aligned}
\frac{\partial[\text{Cyclin B}]}{\partial t} &= D\nabla^2[\text{Cyclin B}] \\
&\quad + k_{\text{synth}} - k_{\text{dest}}[\text{APC}]_{\text{act}}[\text{Cyclin B}] - k_a\left([\text{Cdk1}]_{\text{tot}}\right. \\
&\quad - [\text{Cyclin B-Cdk1}] - [\text{Cyclin B-Cdk1-Yp}] \\
&\quad \left. - [\text{Cyclin B-Cdk1-YpTp}] - [\text{Cyclin B-Cdk1-Tp}]\right)[\text{Cyclin B}] \\
&\quad + k_d[\text{Cyclin B-Cdk1}] \\
\frac{\partial[\text{Cyclin B-Cdk1}]}{\partial t} &= D\nabla^2[\text{Cyclin B-Cdk1}] \\
&\quad + k_a\left([\text{Cdk1}]_{\text{tot}} - [\text{Cyclin B-Cdk1}] - [\text{Cyclin B-Cdk1-Yp}]\right. \\
&\quad \left. - [\text{Cyclin B-Cdk1-YpTp}] - [\text{Cyclin B-Cdk1-Tp}]\right)[\text{Cyclin B}] \\
&\quad - k_d[\text{Cyclin B-Cdk1}] - k_{\text{dest}}[\text{APC}]_{\text{act}}[\text{Cyclin B-Cdk1}]
\end{aligned}
$$

$$
\begin{aligned}
& -k_{\text{wee1}}[\text{Wee1}]_{\text{act}}[\text{Cyclin B-Cdk1}] \\
& -k_{\text{wee1,basal}}\left([\text{Wee1}]_{\text{tot}}-[\text{Wee1}]_{\text{act}}\right)[\text{Cyclin B-Cdk1}] \\
& +k_{\text{cdc25}}[\text{Cdc25}]_{\text{act}}[\text{Cyclin B-Cdk1-Yp}] \\
& +k_{\text{cdc25,basal}}\left([\text{Cdc25}]_{\text{tot}}-[\text{Cdc25}]_{\text{act}}\right)[\text{Cyclin B-Cdk1-Yp}]
\end{aligned}
$$

$$
\begin{aligned}
\frac{\partial[\text{Cyclin B-Cdk1-Yp}]}{\partial t} = {} & D\nabla^2[\text{Cyclin B-Cdk1-Yp}] \\
& +k_{\text{wee1}}[\text{Wee1}]_{\text{act}}[\text{Cyclin B-Cdk1}] \\
& +k_{\text{wee1,basal}}\left([\text{Wee1}]_{\text{tot}}-[\text{Wee1}]_{\text{act}}\right)[\text{Cyclin B-Cdk1}] \\
& -k_{\text{cdc25}}[\text{Cdc25}]_{\text{act}}[\text{Cyclin B-Cdk1-Yp}] \\
& -k_{\text{cdc25,basal}}\left([\text{Cdc25}]_{\text{tot}}-[\text{Cdc25}]_{\text{act}}\right)[\text{Cyclin B-Cdk1-Yp}] \\
& -k_{\text{cak}}[\text{Cyclin B-Cdk1-Yp}]+k_{\text{pp2c}}[\text{Cyclin B-Cdk1-YpTp}] \\
& -k_{\text{dest}}[\text{APC}]_{\text{act}}[\text{Cyclin B-Cdk1-Yp}]
\end{aligned}
$$

$$
\begin{aligned}
\frac{\partial[\text{Cyclin B-Cdk1-YpTp}]}{\partial t} = {} & D\nabla^2[\text{Cyclin B-Cdk1-YpTp}] \\
& +k_{\text{cak}}[\text{Cyclin B-Cdk1-Yp}]-k_{\text{pp2c}}[\text{Cyclin B-Cdk1-YpTp}] \\
& -k_{\text{cdc25}}[\text{Cdc25}]_{\text{act}}[\text{Cyclin B-Cdk1-YpTp}] \\
& -k_{\text{cdc25,basal}}\left([\text{Cdc25}]_{\text{tot}}-[\text{Cdc25}]_{\text{act}}\right)[\text{Cyclin B-Cdk1-YpTp}] \\
& +k_{\text{wee1}}[\text{Wee1}]_{\text{act}}[\text{Cyclin B-Cdk1-Tp}] \\
& +k_{\text{wee1,basal}}\left([\text{Wee1}]_{\text{tot}}-[\text{Wee1}]_{\text{act}}\right)[\text{Cyclin B-Cdk1-Tp}] \\
& -k_{\text{dest}}[\text{APC}]_{\text{act}}[\text{Cyclin B-Cdk1-YpTp}]
\end{aligned}
$$

$$
\begin{aligned}
\frac{\partial[\text{Cyclin B-Cdk1-Tp}]}{\partial t} = {} & D\nabla^2[\text{Cyclin B-Cdk1-Tp}] \\
& +k_{\text{cdc25}}[\text{Cdc25}]_{\text{act}}[\text{Cyclin B-Cdk1-YpTp}] \\
& +k_{\text{cdc25,basal}}\left([\text{Cdc25}]_{\text{tot}}-[\text{Cdc25}]_{\text{act}}\right)[\text{Cyclin B-Cdk1-YpTp}] \\
& -k_{\text{wee1}}[\text{Wee1}]_{\text{act}}[\text{Cyclin B-Cdk1-Tp}] \\
& -k_{\text{wee1,basal}}\left([\text{Wee1}]_{\text{tot}}-[\text{Wee1}]_{\text{act}}\right)[\text{Cyclin B-Cdk1-Tp}] \\
& -k_{\text{dest}}[\text{APC}]_{\text{act}}[\text{Cyclin B-Cdk1-Tp}]
\end{aligned}
$$

$$
\begin{aligned}
\frac{\partial[\text{Cdc25}]_{\text{act}}}{\partial t} = {} & D\nabla^2[\text{Cdc25}]_{\text{act}} \\
& +k_{\text{cdc25,on}}\left(\frac{[\text{Cyclin B-Cdk1-Tp}]^{n_{\text{cdc25}}}}{(EC50)_{\text{cdc25}}^{n_{\text{cdc25}}}+[\text{Cyclin B-Cdk1-Tp}]^{n_{\text{cdc25}}}}\right) \\
& \times\left([\text{Cdc25}]_{\text{tot}}-[\text{Cdc25}]_{\text{act}}\right)-k_{\text{cdc25,off}}[\text{Cdc25}]_{\text{act}}
\end{aligned}
$$

$$
\begin{aligned}
\frac{\partial[\text{Wee1}]_{\text{act}}}{\partial t} = {} & D\nabla^2[\text{Wee1}]_{\text{act}} \\
& -k_{\text{wee1,off}}\left(\frac{[\text{Cyclin B-Cdk1-Tp}]^{n_{\text{wee1}}}}{(EC50)_{\text{wee1}}^{n_{\text{wee1}}}+[\text{Cyclin B-Cdk1-Tp}]^{n_{\text{wee1}}}}\right) \\
& \times[\text{Wee1}]_{\text{act}}+k_{\text{wee1,on}}\left([\text{Wee1}]_{\text{tot}}-[\text{Wee1}]_{\text{act}}\right)
\end{aligned}
$$

$$\frac{\partial[\mathrm{Plx1}]_{\mathrm{act}}}{\partial t} = D\nabla^2[\mathrm{Plx1}]_{\mathrm{act}} + k_{\mathrm{plx1,on}}\left(\frac{[\text{Cyclin B-Cdk1-Tp}]^{n_{\mathrm{plx1}}}}{(EC50)_{\mathrm{plx1}}^{n_{\mathrm{plx1}}} + [\text{Cyclin B-Cdk1-Tp}]^{n_{\mathrm{plx1}}}}\right) \times ([\mathrm{Plx1}]_{\mathrm{tot}} - [\mathrm{Plx1}]_{\mathrm{act}}) - k_{\mathrm{plx1,off}}[\mathrm{Plx1}]_{\mathrm{act}}$$

$$\frac{\partial[\mathrm{APC}]_{\mathrm{act}}}{\partial t} = D\nabla^2[\mathrm{APC}]_{\mathrm{act}} + k_{\mathrm{APC,on}}\left(\frac{[\mathrm{Plx1}]_{\mathrm{act}}^{n_{\mathrm{APC}}}}{(EC50)_{\mathrm{plx1}}^{n_{\mathrm{APC}}} + [\mathrm{Plx1}]_{\mathrm{act}}^{n_{\mathrm{APC}}}}\right) \times ([\mathrm{APC}]_{\mathrm{tot}} - [\mathrm{APC}]_{\mathrm{act}}) - k_{\mathrm{APC,off}}[\mathrm{APC}]_{\mathrm{act}}$$

In our simulations, each of the densities ([Cyclin B], [Cyclin B-Cdk1], [Cyclin B-Cdk1-Yp], [Cyclin B-Cdk1-YpTp], [Cyclin B-Cdk1-Tp], $[\mathrm{Cdc25}]_{\mathrm{act}}$, $[\mathrm{Wee1}]_{\mathrm{act}}$, $[\mathrm{Plx1}]_{\mathrm{act}}$, $[\mathrm{APC}]_{\mathrm{act}}$) are functions of both space and time. For the parameters, all concentrations are in arbitrary units (au), all times are in sec, and all rates are either in $(\mathrm{sec})^{-1}$ or $(\mathrm{au} \times \mathrm{sec})^{-1}$. Except where noted, the parameters are as follows:

$$\begin{aligned}
k_{\mathrm{synth}} &= 0.02 \\
r &= 10 \\
D &= 0.001 \\
k_{\mathrm{dest}} &= 0.01 \\
k_a &= 0.1 \\
k_d &= 0.001 \\
k_{\mathrm{wee1}} &= 0.05 \\
k_{\mathrm{wee1,basal}} &= k_{\mathrm{wee1}}/r \\
k_{\mathrm{cdc25}} &= 0.1 \\
k_{\mathrm{cdc25,basal}} &= k_{\mathrm{cdc25}}/r \\
[\mathrm{Cyclin}]_{\mathrm{tot}} &= 230 \\
[\mathrm{Cdc25}]_{\mathrm{tot}} &= 15 \\
[\mathrm{Wee1}]_{\mathrm{tot}} &= 15 \\
[\mathrm{APC}]_{\mathrm{tot}} &= 50 \\
[\mathrm{Plx1}]_{\mathrm{tot}} &= 50 \\
n_{\mathrm{wee1}} &= 4 \\
n_{\mathrm{cdc25}} &= 4 \\
n_{\mathrm{APC}} &= 4 \\
n_{\mathrm{plx1}} &= 4 \\
(EC50)_{\mathrm{plx1}} &= 40
\end{aligned}$$

$$
\begin{aligned}
(EC50)_{\text{wee1}} &= 40 \\
(EC50)_{\text{cdc25}} &= 40 \\
\text{APC} &= 40 \\
k_{\text{cdc25,on}} &= 1.75 \\
k_{\text{cdc25,off}} &= 0.2 \\
k_{\text{APC,on}} &= 1 \\
k_{\text{APC,off}} &= 0.15 \\
k_{\text{plx1,on}} &= 1 \\
k_{\text{plx1,off}} &= 0.15 \\
k_{\text{wee1,on}} &= 0.2 \\
k_{\text{wee1,off}} &= 1.75 \\
k_{\text{cak}} &= 0.8 \\
k_{\text{pp2c}} &= 0.008
\end{aligned}
$$

Besides APC, the cell-cycle proteins have very similar molecular weights (listed below). Treating proteins as spheres, the radius of the protein r scales as $M^{1/3}$, where M is the mass of the protein. From the Stokes-Einstein relation, the diffusion constant (D) in low Reynolds number liquids scales as r^{-1}. Based on the Stokes-Einstein relation, a two-fold increase in the mass of the protein reduces D by 20%, and a five-fold increase in the protein mass reduces D by 40%. Thus, we set all diffusion constants to be equal and within the range of *in vivo* measurements for our simulations. We also note that D can be affected by specific or non-specific *in vivo* interactions.

Table S1: Molecular weights of cell-cycle proteins

Species	MW (kDa)
Cyclin B-Cdk1	79.4
Wee1	67
Cdc25	60.3
Plx1	68.13
Cyclin B	44.92
Cdk1	34.52
APC	300

References

[1] Pomerening J. R., Kim S. Y., and Ferrell J.E. (2005) Systems-level Dissection of the Cell Cycle Oscillator: Bypassing Positive Feedback Produces Damped Oscillations. Cell **122** 565-578.

[2] Hartley R. S., Rempel R. E., and Maller J.L. (1996) In vivo regulation of the early embryonic cell cycle in Xenopus. *Developmental Biology* **173** 408-419.

[3] Hershko A., Ganoth D., Sudakin V., Dahan A., Cohen L.H., *et al.* (1994) Components of a system that ligates cyclin to ubiquitin and their regulation by the protein kinase cdc2. Journal of Biological Chemistry **269** 4940-4946.

[4] King R.W., Peters J., Tugendreich S., Rolfe M., Hieter P., *et al.* (1995) A 20S complex containing CDC27 and CDC16 catalyzes the mitosis-specific conjugation of ubiquitin to cyclin B. Cell, **81** 279-288.

[5] Peters J.M. (2002) The anaphase-promoting complex: Proteolysis in mitosis and beyond. Molecular Cell **9** 931-943.
